# Scalable High-Precision Trimming of Photonic Resonances by Polymer Exposure to Energetic Beams

**DOI:** 10.1021/acs.nanolett.3c00220

**Published:** 2023-05-17

**Authors:** Nikolaos Farmakidis, Hao Yu, June Sang Lee, Johannes Feldmann, Mengyun Wang, Yuhan He, Samarth Aggarwal, Bowei Dong, Wolfram H. P. Pernice, Harish Bhaskaran

**Affiliations:** † Department of Materials, University of Oxford, Parks Road, Oxford OX1 3PH, U.K.; ‡ Kirchhoff-Institute for Physics, Heidelberg University, 69120 Heidelberg, Germany

**Keywords:** photonic integrated circuits, tunable photonics, nanofabrication

## Abstract

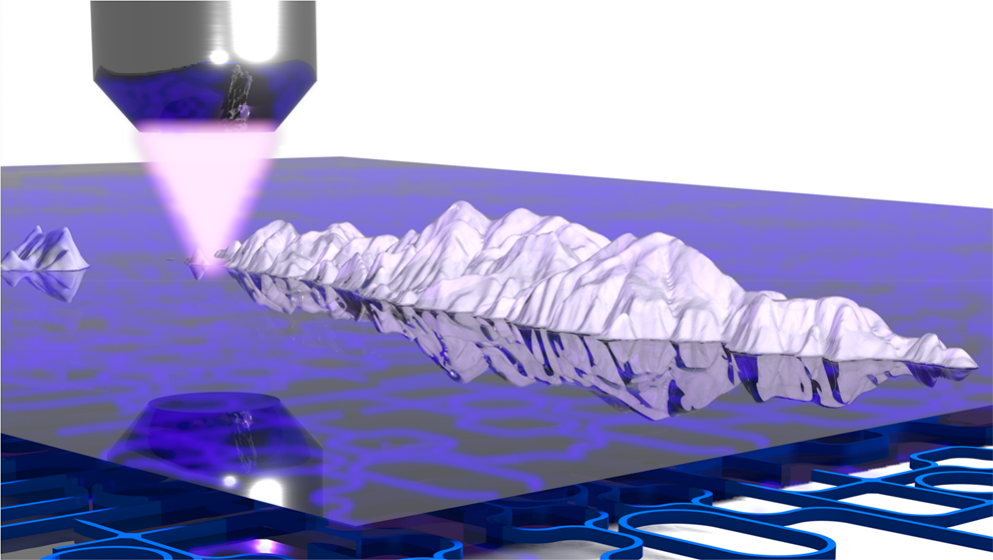

Integrated photonic circuits (PICs) have seen an explosion in interest, through to commercialization in the past decade. Most PICs rely on sharp resonances to modulate, steer, and multiplex signals. However, the spectral characteristics of high-quality resonances are highly sensitive to small variations in fabrication and material constants, which limits their applicability. Active tuning mechanisms are commonly employed to account for such deviations, consuming energy and occupying valuable chip real estate. Readily employable, accurate, and highly scalable mechanisms to tailor the modal properties of photonic integrated circuits are urgently required. Here, we present an elegant and powerful solution to achieve this in a scalable manner during the semiconductor fabrication process using existing lithography tools: by exploiting the volume shrinkage exhibited by certain polymers to permanently modulate the waveguide’s effective index. This technique enables broadband and lossless tuning with immediate applicability in wide-ranging applications in optical computing, telecommunications, and free-space optics.

Interest in optical technologies has experienced an extraordinary surge in recent decades due to the multifaceted functionality that optical signals enable. A proliferation of both integrated and free-space components have emerged capitalizing on the speed, bandwidth, and functionality of photonics to realize technologies ranging from displays^1,2^ to beam steering and routing,^3−5^ optical data storage,^6−12^ computation,^13−15^ and sensing. Resonances in photonic nanostructures such as microring resonators form the backbone for many of these applications and provide high sensitivity in their operation.^16−18^ However, the high quality factor resonances employed come at the cost of stringent design tolerances which are not typically satisfied in their fabrication.^17−19^ Active electrical components such as thermo-optic^20,21^ or electro-optic^22,23^ phase shifters are therefore commonly employed; these are, however, power hungry and occupy valuable chip real estate.

One route to addressing this significant challenge is through the use of materials which show a permanent modulation of their optical properties^24−27^ or alternatively using materials which demonstrate permanent geometrical or structural changes based on external programming^28−30^ between binary or multiple states. This can be achieved using electrical, optical, or thermal stimuli which can permanently modify the atomic arrangement in the material or induce permanent chemical changes.^18^

In this work, we exploit the property of certain polymers to undergo chain scission and crosslinking upon exposure to energetic beams—both ultraviolet (UV) and electron beams. We unequivocally demonstrate that chain scission and crosslinking induce a volumetric change in the polymer film, rather than a modification in its refractive index.^31^ By employing an inexpensive, fab-compatible, and widely available polymer, poly(methyl methacrylate) (PMMA), as an overcladding in photonic circuits, we demonstrate that we can permanently modify the modal properties of the waveguides with excellent control. We demonstrate that this method has considerable strengths in scalability, reproducibility, and compatibility with industrial processes, which make it highly applicable to standard foundry wafer processing. Our experiments shed light on the mechanism of this modification, and using this we are able to accurately predict the propagation characteristics of PMMA-clad waveguides.^32,33^ Our technique demonstrates high-resolution trimming of single- and multi-resonator photonic circuits in excess of 1 nm with picometer precision.

The mechanism for modulating the topography of the polymer is illustrated in Figure 1a. An energetic electron or ultraviolet (UV) optical beam is employed to expose the polymer and induce chain scission.^34^ This process is known to cause the outgassing of volatile species (CO_2_, CH_4_, and more), which locally reduces the volume of the polymer.^35^ Localized changes in topography can then be observed directly by measuring the topography of the film using an atomic force microscope (AFM) (Figure 1c) or indirectly through color changes resulting from modifications in the Fabry–Perot cavity of the dielectric stack (Figure 1b).^31^Figure 1d shows a linear reduction in the thickness (Δ*H*) of the stack with increasing exposure dose as measured by AFM for different polymer thicknesses. The initial thickness of the polymer here is controlled by changing the initial concentration of the polymer during spin coating. A comparison between the height changes and color changes with increasing dose is shown in Figures 1b,c. Here PMMA 495k in anisole at four different concentrations (2%, 4%, 6%, and 8%) has been exposed with increasing dosage from left to right and from top to bottom between 0 and 3000 μC/cm^2^ (details are given in Methods). At low doses, chain scission dominates, which causes a volume reduction, evidenced as depressions in the polymer film. The total height reduction Δ*H* is found to scale linearly with the initial thickness of the polymer, producing a change of ∼10% of the thickness *H*. At high dosage, the polymer chains begin to crosslink, which is evidenced by a phase inversion and a local increase in the height of the PMMA film. The color changes observed in the thin film resulting from the modification of the Fabry–Perot cavity can be seen in Figure 1e, which agree well with the simulated spectra for modifications in Δ*Η*/*H* = 0–10% in the absence of any modifications in the optical properties of the polymer.

**Figure 1:**
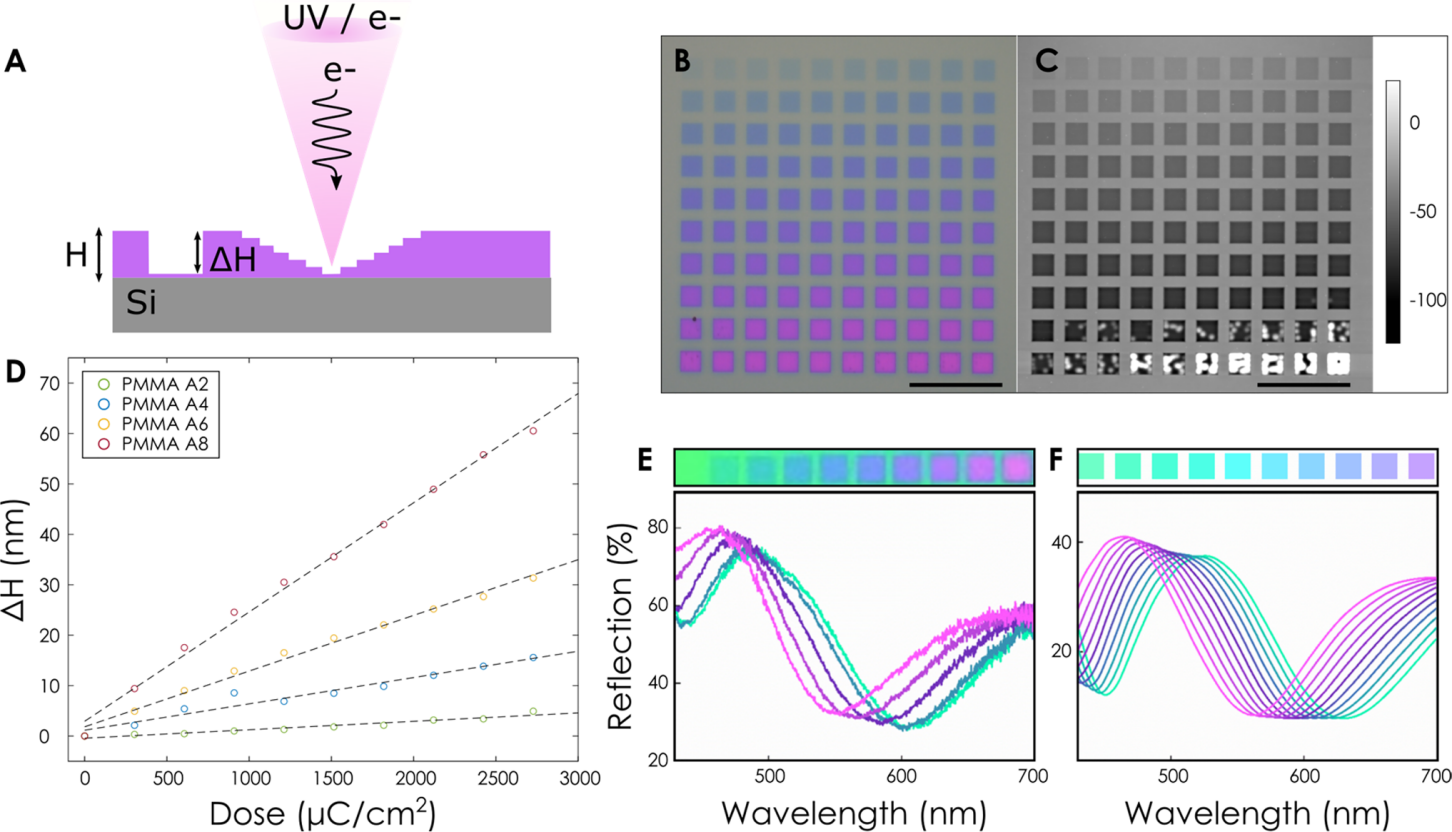
Grayscale topography changes due to chain scission in PMMA. (A) Schematic of height modulation with exposure to energetic electron or photon beams. (B, C) Optical and atomic force microscope (AFM) micrographs of individual pixels written on a film of PMMA with an 8% concentration with increasing dose between 0 and 3000 μC/cm^2^ (dose increases from left to right and top to bottom). (D) Reduction in height upon exposure for four concentrations of PMMA 495k. (E, F) Experimental and simulated color pixels for PMMA 495k on silicon with Δ*Η*/*H* = 0–10%. Scale bars are 10 μm.

To decouple any optical changes which could be simultaneously occurring in the film, we directly measure the optical parameters of exposed films by ellipsometry. As evidenced in Figure 2a, the refractive index of the polymer remains unchanged beyond the visible wavelength range (0.8–1.6 μm) with a refractive index (*n*) of 1.5 and near zero changes in the extinction coefficient (*k*) (Figure S1). We have thus proved that any optical effects are dominated by the structural changes in the polymer rather than modifications in its dielectric function.

**Figure 2:**
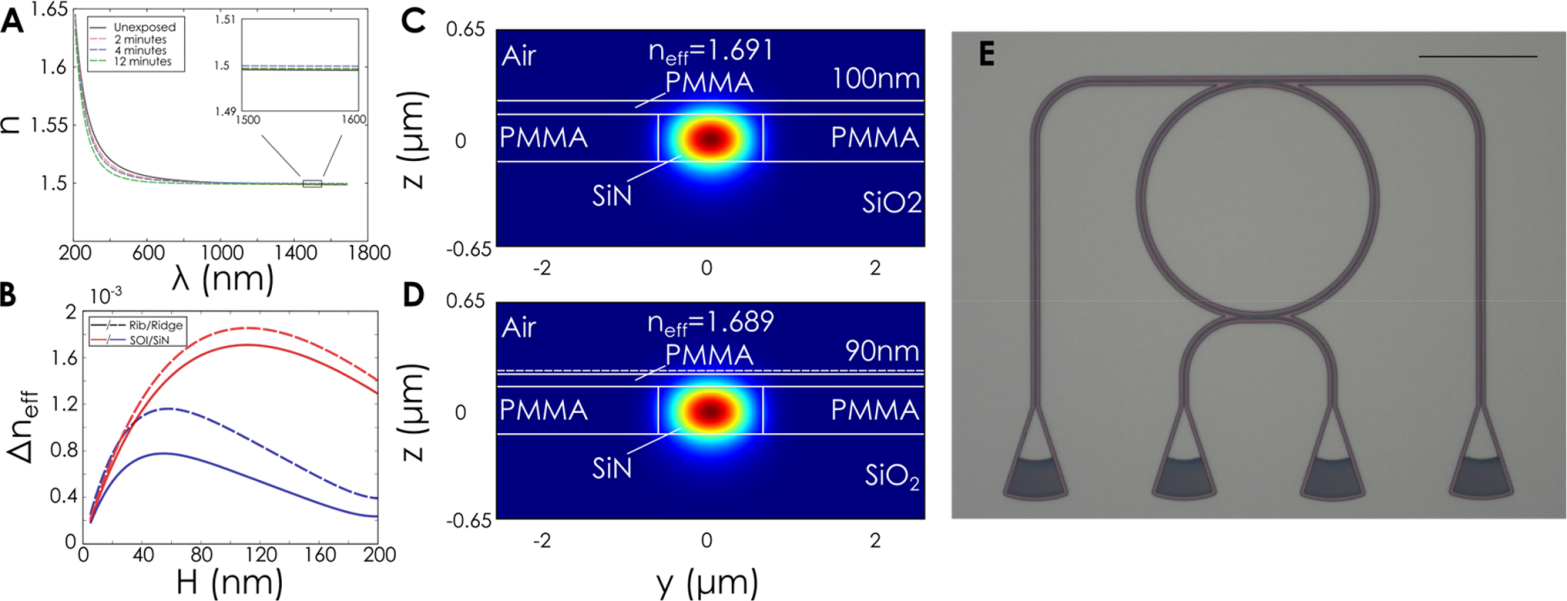
Modulation of the mode effective index in photonic waveguides due to a reduction in the polymer cladding thickness. (A) Ellipsometry measurements on PMMA thin films at different exposure doses. The results show no changes in the refractive index of the film at telecommunications wavelengths. (B) Simulated mode effective index changes for different waveguide geometries as a function of the initial thickness of PMMA. Here a 10% change in height is used. (C, D) Simulated mode in the cross-section of a PMMA-cladded waveguide for PMMA thicknesses of 100 and 90 nm, respectively. The waveguide remains lossless while the effective index of the mode is modulated. (E) Optical micrograph of fabricated add-drop ring resonator. The scale bar is 100 μm.

We then exploit this capability by modulating the modal characteristics of photonic integrated circuits. In order to determine the cladding thickness which produces the strongest modulation in the effective index of the propagating mode, we perform mode simulations on silicon (SOI) and silicon nitride (SiN) PICs for varying polymer thicknesses. Here, the electric field of the optical mode of the waveguide decays exponentially throughout the polymer cladding and an optimal balance between the interaction volume and the distance to the waveguide is sought to maximize the effect of the polymer shrinkage. This is illustrated in Figure 2b. where for a height modulation Δ*H*/*H* = 10% the change in effective index between the two states shows a maximum at an initial thickness of 50 nm for silicon and 100 nm for silicon nitride based waveguides. This is attributed to the higher mode confinement in Si compared to SiN.

In Figure 2c,d the cross-section of the mode propagating inside a SiN ridge waveguide is plotted. The waveguide is cladded with a layer of 100 nm PMMA which corresponds to the maximum Δ*N*_eff_ in Figure 2b. The effective index of the mode is found to be 1.691 with near-zero propagation loss. The thickness of the PMMA is subsequently reduced to 90 nm (a reduction of 10% in the thickness) and is found to remain lossless with a mode effective index of 1.689, producing a change of Δ*N*_eff_ = 1.85 × 10^–3^.

## Add-Drop Ring Trimming

**Figure 3:**
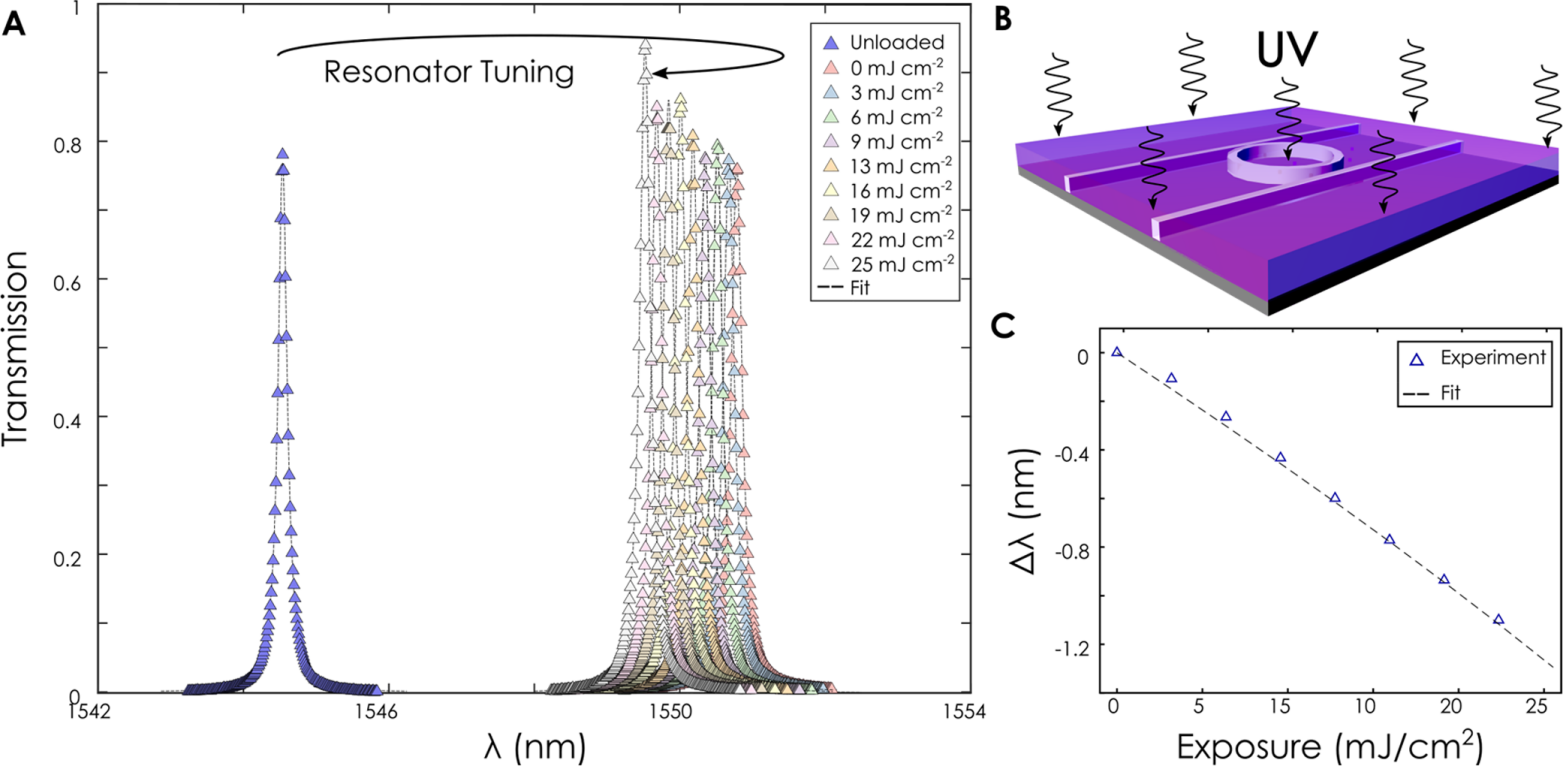
Resonance tuning of a photonic ring resonator. (A) An as-fabricated resonator (left/blue) is coated with PMMA and the resonance is red-shifted. Subsequent sequential exposure of the PMMA-cladded ring causes the resonance to blue-shift progressively with exposure dose. (B) Schematic of PMMA-cladded ring resonator exposed to ultraviolet light. (C) Resonance wavelength from (A) as a function of exposure dose. A linear relationship is found within the tuning range.

We proceed to show that the induced change in effective index can be employed to accurately and precisely tune the spectrum of an add-drop ring resonator. Figure 2e shows an as-fabricated ring resonator with a resonance peak at 1545 nm (Figure 3a). Light is guided into the trough port via conventional Bragg grating couplers. Here the presence of PMMA as an overcladding has a negligible effect on the coupling performance of the Bragg gratings due to its low refractive index, which lies in the vicinity of the SiO_2_ substrate index values. Upon coating with a layer of PMMA, the resonance of the ring is measured to red-shift to 1551 nm due to the presence of the polymer cladding. Figure 3a shows the ring spectrum in the as-fabricated state as well as the red-shifted peak of the as-deposited polymer. Sequential exposure of the polymer film with a UV source continuously blue-shifts the resonance of the ring by more than 1 nm or approximately half the ring’s FSR. Figure 3c shows stable linear tuning of the resonance wavelength of the ring with exposure dose by which the resonance of any ring can be precisely tuned, with no detectable change in the quality factor of the resonances (Figure S1). This is achieved using a uniform coating of the chip in its entirety and a flood exposure to UV light.

## Multi-Ring Trimming

**Figure 4:**
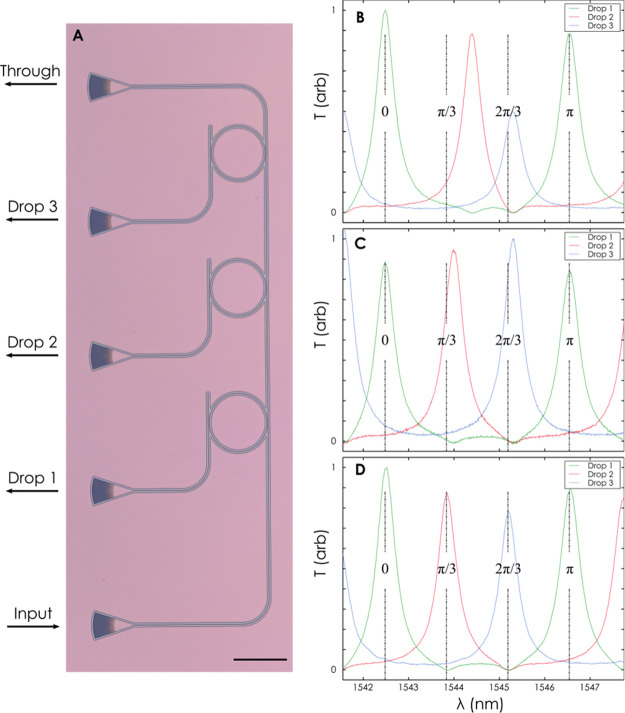
Resonance tuning of a cascaded ring resonator system. A local UV exposure was performed on resonators 2 and 3 for a total of 400 and 70 s, respectively. (A) Optical micrograph of three cascaded ring resonators. (B–D) Achieving equally spaced resonances by locally exposing each ring. (B) As-fabricated rings with a polymer cladding. (C, D) Progressive exposure of the ring resonators to achieve equally spaced resonances in (D). The scale bar is 100 μm.

We proceed to demonstrate the scalability of the technique and addressability of multi-component photonic circuits by individually trimming the resonance wavelength of three cascaded ring resonators. Figure 4a shows a three-resonator cascaded system with rings of 50 μm radius. In the as-fabricated state as well as in the as-deposited states, the spectral positions of the resonances are randomly distributed, caused by inevitable fabrication errors (Figure 4b). We subsequently perform a two-step exposure on each polymer-cladded ring individually to achieve equally spaced peaks within one FSR. Here the first resonator remained unexposed throughout the experiment while the second resonator received a single initial UV exposure for 290 s and was remeasured. Using a second exposure for 110 s on the second resonator and 70 s on the third resonator, the locations of the peaks were precisely tuned to one-third and two-thirds of the FSR, respectively. Figure 4c shows that the peak of the second resonator has been coarsely tuned to approximately one-third of the FSR (red curve), while in Figure 4d both resonators have been trimmed to precisely one-third (red curve) and two-thirds (green curve) of the FSR, respectively. The spectra of the resonators are collected by injecting light into the input port (Figure 4a and Figure S3) and subsequently measuring each resonator via drop ports 1, 2, and 3. The progressive trimming is shown in Figures 4b–d, and the total transmission of the bus waveguide is shown in Figure S2.

## 3D Nanofabrication

**Figure 5:**
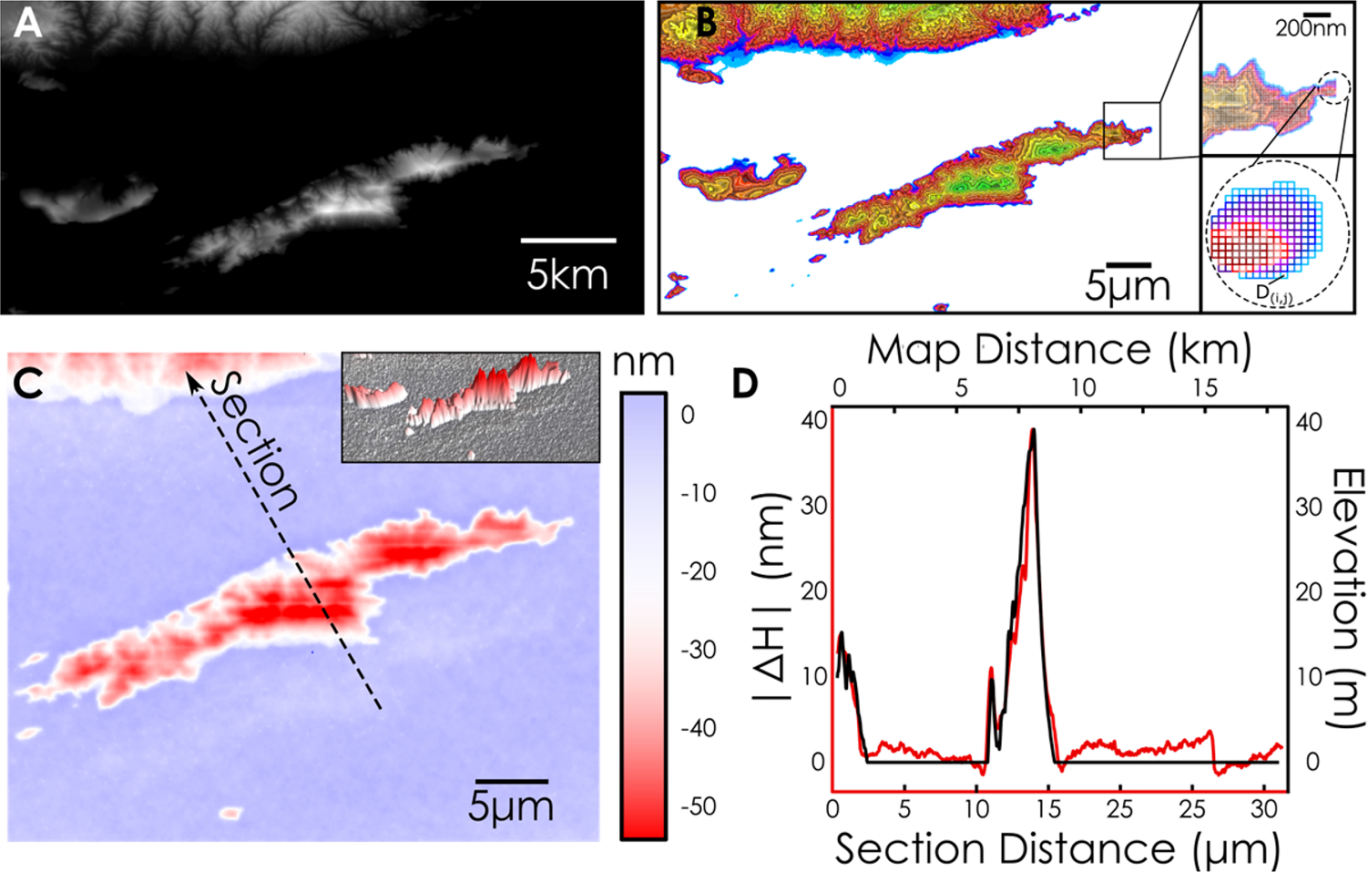
Grayscale nanolithography using dose modulation on PMMA. (A–D) Replication of the three-dimensional landscape of the Greek island Hydra in 3D using dose modulation. The landscape in (A) is converted to a grayscale image and fractured to 100 linearly spaced dose pixels in (B). (C, D) AFM scan of the exposed area and height section showing excellent agreement between the original image and the fabricated replica with an in-plane resolution of approximately 50 nm.

It has been demonstrated that high-quality resonances in integrated photonics can be modulated by locally modifying the height of a polymer overcladding which in effect utlizes a uniform modulation of the structure out of plane. However, as the polymer layer also affords nanoscale control in plane, we proceed to demonstrate this capability by structuring the polymer film in three dimensions for the production of complex nanostructures which can be employed in photonics and beyond. Here, by precisely modulating the exposure dose in space, the topography of the polymer can be modulated with sub-nanometer precision. The topography produced can then be used directly for three-dimensional modifications of the optical properties of micro- and nanostructures (e.g., metasurfaces, multimode couplets) and can be even employed as a fabrication mold for further processing. To demonstrate this capability, we reproduced the complex 3D landscape of the Greek island Hydra shown in Figure 5a directly onto the polymer. The topography was discretized to 100 different levels according to the height image (Figure 5a), reduced to 1:10^9^ scale,and mapped to the height changes (Δ*H*) reported in Figure 1D. Each of these discrete levels are subsequently fractured and converted into a design file as shown in Figure 5B and exposed with linearly spaced doses between 0 and 3000 μC/cm^2^ to produce a replica of the original topography at a 1:10^9^ scale. The topography of the fabricated replica is shown in the AFM micrograph of Figure 5C Additionally, a comparison between a section of the original landscape image and the replicated topography is shown in Figure 5D wherein the two are in excellent agreement. The three-dimensional structures produced can be further transferred to flexible substrates as shown in Figures S4 and S5.

## Conclusion

We have demonstrated the implementation of a scalable, high-resolution, and lossless trimming mechanism for photonic circuits using a single-shot controlled exposure of the photosensitive polymer PMMA. In contrast to previous works attributing modifications in the optical mode propagation to a modulation in the refractive index of the material, we show that the refractive index remains largely unchanged while the topography of the film is modified. By understanding and modeling such changes, we show a single-shot linear tuning range of over 1 nm, with picometer precision. We further demonstrate individual trimming of cascaded ring resonators to produce equally spaced resonances without incurring additional optical losses, which is highly desired in large-scale integrated photonic circuits. Owing to the ultrahigh out-of-plane resolution afforded by this method, we extend this work to the patterning of complex three-dimensional structures with high resolution and fidelity, showing the potential of this technique in a broad range of applications ranging from metasurfaces and free-space optics to integrated nanophotonics.

## Methods

### Sample Preparation

Solutions of PMMA 495k in anisole were prepared in concentrations ranging from 8% (A8) to 2% (A2). Intrinsically doped silicon wafers were used for all samples with the exception of samples including photonic integrated circuits, which relied on the silicon nitride on insulator (SiN) platform. Samples were cleaned in acetone and IPA, dried using nitrogen, subsequently baked at 150 °C to remove water condensation, and treated with O_2_ plasma for 1 min. The samples were subsequently spin-coated with different thicknesses of PMMA and baked at 180 °C for 10 min to remove all solvents from the thin film.

### Fabrication of Photonic Integrated Circuits

Silicon nitride on insulator wafers with a 330 nm silicon nitride layer were purchased from Rogue Valley Microdevices and were cleaned according to the procedure outlined above. Samples were subsequently coated with CSAR65 positive EBL resist and patterned in a Jeol JBX5500 system. The samples were finally etched by CHF_3_ dry chemical etching to create the waveguides, grating couplers, and ring resonators. Finally the resist was removed and the wafers were treated according to the procedure outlined in the previous section before being exposed to UV radiation using a SUSS mask aligner with a power of 8.49 mW/cm^2^ measured at 240 nm wavelengths.

## Data Availability

All data needed to evaluate the conclusions in the paper are present in the paper and/or the Supporting Information.

## Supporting Information Available

Quality factor measurement of the polymer-cladded ring, through-port spectrum before and after trimming, optical micrograph of the ring resonator, transfer of three-dimensional structures to a flexible substrate, and complex pattern transfer to PDMS (PDF)
