# Correction to “Scalable High-Precision Trimming of Photonic Resonances by Polymer Exposure to Energetic Beams”

**DOI:** 10.1021/acs.nanolett.4c03363

**Published:** 2024-10-01

**Authors:** Nikolaos Farmakidis, Hao Yu, June Sang Lee, Johannes Feldmann, Mengyun Wang, Yuhan He, Samarth Aggarwal, Bowei Dong, Wolfram HP Pernice, Harish Bhaskaran

The full citation of the original article is given in the References section below.^1^

In Figure 2b, in the second entry of the legend there was a color mismatch which we have now remedied.

**Figure 2:**
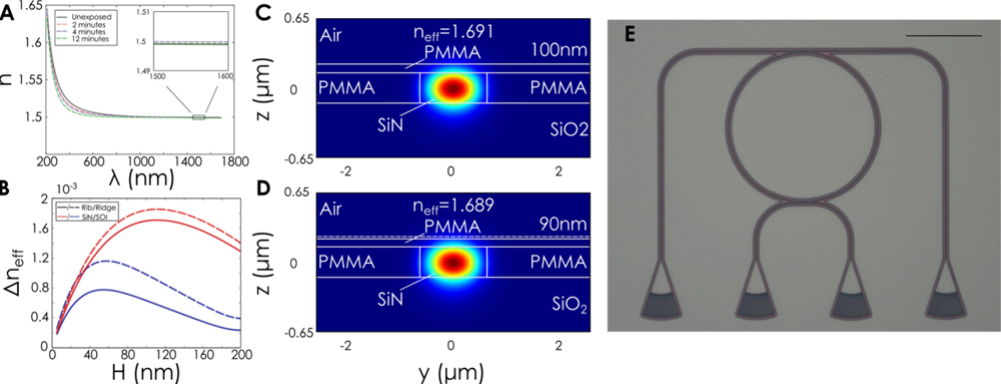
Modulation of the mode effective index in photonic waveguides due to a reduction in the polymer cladding thickness. **(A)** Ellipsometry measurements on PMMA thin films at different exposure doses. Results show no changes in the refractive index of the film at telecommunications wavelengths. **(B)** Simulated mode effective index changes for different waveguide geometries as a function of the initial thickness of PMMA. Here a 10% change in height is used. **(C**, **D)** Simulated mode in the cross-section of a PMMA-cladded waveguide for a PMMA thickness of 100 and 90 nm, respectively. The waveguide remains lossless while the effective index of the mode is modulated. **(E)** Optical micrograph of fabricated add-drop ring resonator. Scale bar is 100 μm.

We identified an error in the x-axis of Figure S1 where the axis should be minutes instead of seconds as described correctly in the main text. This conversion error also affects the x-axis of Figure 3C and the legend entries of Figure 3A which should be J·cm^–2^ not mJ·cm^–2^.

**Figure 3:**
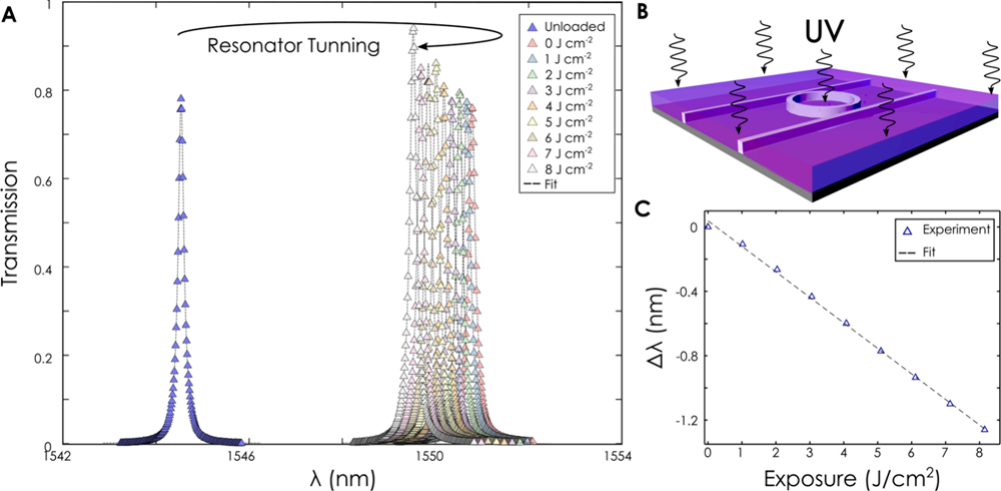
Resonance tuning of a photonic ring resonator. **(A)** An as-fabricated resonator (left/blue) is coated with PMMA and the resonance is red-shifted. Subsequent exposure of the PMMA-cladded ring causes the resonance to blueshift progressively with exposure dose. **(B)** Schematic of PMMA-cladded ring resonator exposed to ultraviolet light. **(C)** Resonance wavelength from **(A)** as a function of exposure dose. A linear relationship is found within the tuning range.

**Figure S1:**
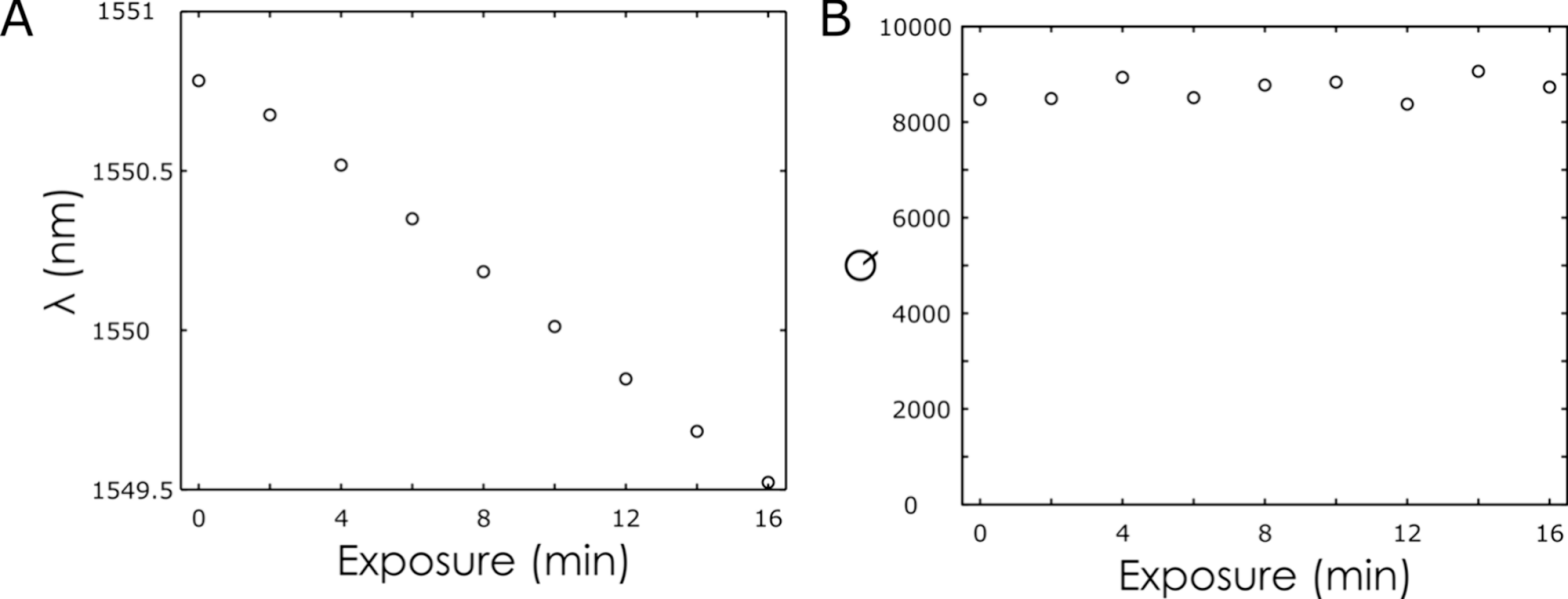
Progressive exposure of a polymer-cladded ring resonator to ultraviolet light. The resonance wavelength of the resonator (A) is shifted while the quality factor of the resonance shows small random fluctuations (B).
