# Supplementary material for: Scalable High-Precision Trimming of Photonic Resonances by Polymer Exposure to Energetic Beams
Source: Nano Lett. 2023 May 17;23(11):4800–6. doi: 10.1021/acs.nanolett.3c00220 (PMC10273466; doi:10.1021/acs.nanolett.3c00220)
Supplement: Supplementary file 1 — nl3c00220_si_001.pdf [file nl3c00220_si_001.pdf]

Supplementary Information

For

Scalable high-precision trimming of photonic resonances by polymer exposure to energetic beams

Nikolaos Farmakidis[1],Hao Yu[1], June Sang Lee[1], Johannes Feldmann[1], Mengyun Wang[1], Yuhan He[1], Samarth Aggarwal[1], Bowei Dong[1] ,Wolfram HP Pernice[2], and Harish Bhaskaran[1]*

[1]Department of Materials, University of Oxford, Parks Road, Oxford OX1 3PH, UK.

[2]Kirchhoff-Institut für Physik, Universität Heidelberg, Heidelberg, Germany.

*Corresponding authors: E-mail: *harish.bhaskaran@materials.ox.ac.uk*

S1. Quality factor measurement of the polymer-cladded ring.

As shown in figure S1, when exposing the polymer-cladded ring resonator, the resonance wavelength is modulated linearly with the exposure time. At the same time, the quality factor of the resonance remains constant (Fig. S1B), indicating that the absorption inside the material remains unchanged during the process.

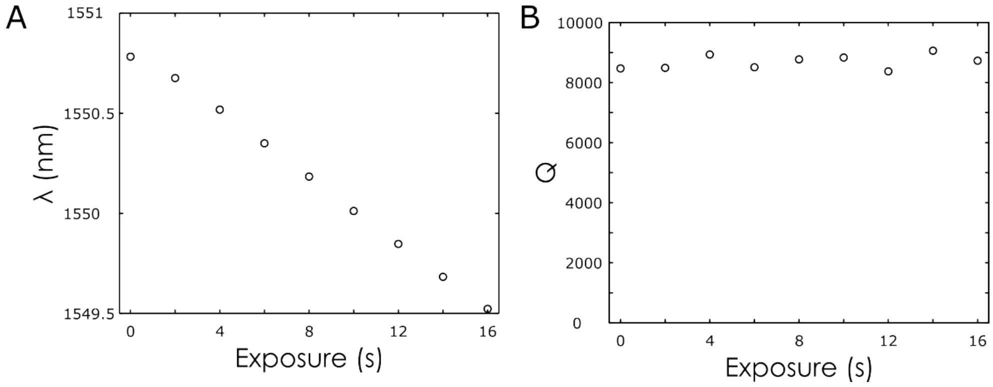

Figure S1: Progressive exposure of a polymer cladded ring resonator to ultra-violet light. The resonance wavelength of the resonator **(A)** is shifted while the quality factor of the resonance shows small random fluctuations (**B**)

S2. Through-port spectrum before and after trimming

Figure S2 shows the spectrum of the multi-ring photonic structure as measured through the common bus waveguide. The red curve, shows the nearly random spectral positions of the three ring depressions before trimming the structure. After trimming the structure by exposing the polymer cladding, the spectrum of the structure (blue curve) shows equally spaced resonances. Here, modifications in the total transmission of the three-port structure are attributed to slight modifications to the critical coupling of the ring resonators in the presence of height-modulated PMMA and more importantly in experimental inconsistencies in re-aligning the photonic structures to the measurement setup post-trimming and maintaining identical chip rotation, light polarisation and coupling position on the grating couplers.

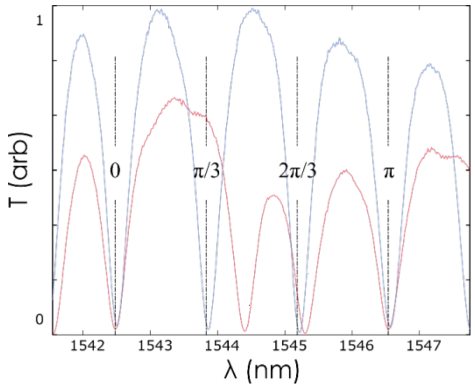

Figure S2: Spectral characteristics of the multi-ring photonic structure before and after the controlled exposure of the polymer. Before trimming (red curve), the resonances are randomly spaced whereas their positions are precisely tuned after the exposure.

S3. Optical image of the ring resonator

Close-up optical micrograph of an add-drop microring resonator. Light is coupled to the center waveguides using the grating couplers shown in figure S3B. Light is coupled to the ring resonator using directional couplers (fig. S3c) and the light output is simultaneously recorded at the through and drop ports.

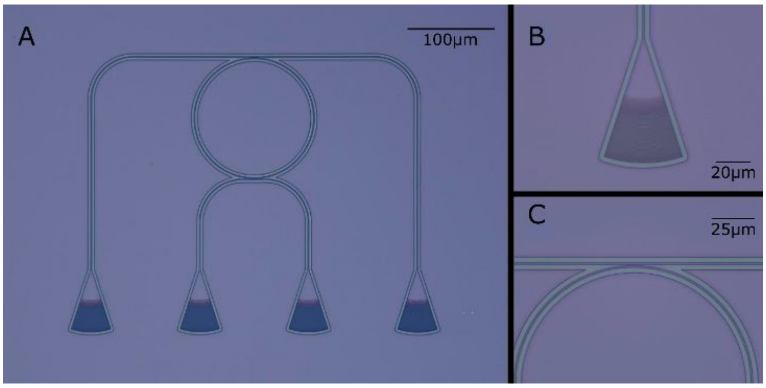

Figure S3: Optical micrographs and close-up views of typical microring resonator. **A**) Single ring microring resonator. **B**) Grating coupler used to couple light to the input waveguides. **C**) Directional coupler used to couple light to the ring.

S4. Transfer of three dimensional structures to a flexible substrate

The three dimensional patterns produced on the polymer film can further serve as a mold. We show that by covering the structures with a curable epoxy, we can transfer the structures to the epoxy when in liquid form and subsequently cure to solidify. Lifting off the initial structures is performed by immersing in acetone.

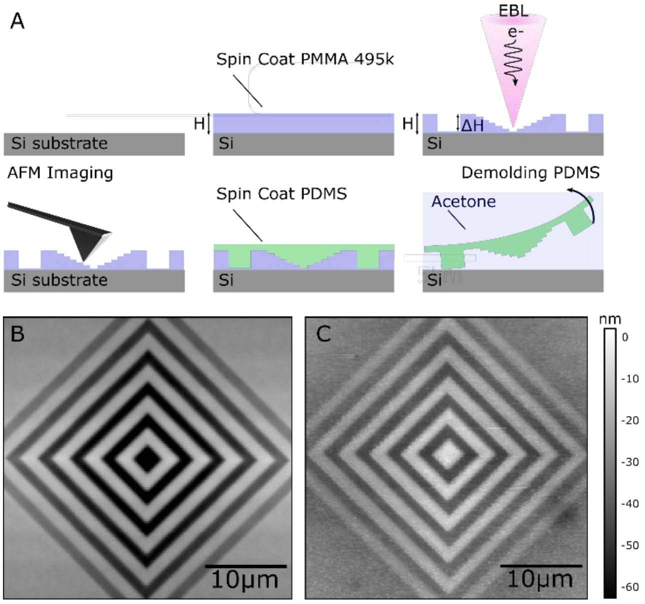

Figure S4: Atomic force micrographs of transferred patterns produced on PMMA to PDMS. **A)** Schematic of transfer process **B)** Initial pattern produced on PMMA **C)** Patterns transferred to PDMS

S5. Complex pattern transfer to PDMS

Highly complex patterns can be transferred to PDMS according to the procedure in figure S4. Here we show that the scaled topography map can be transferred to a PDMS substrate with high fidelity. Figure S5A shows the original image and figure S5B shows the optical image of the transferred patterns to PDMS.

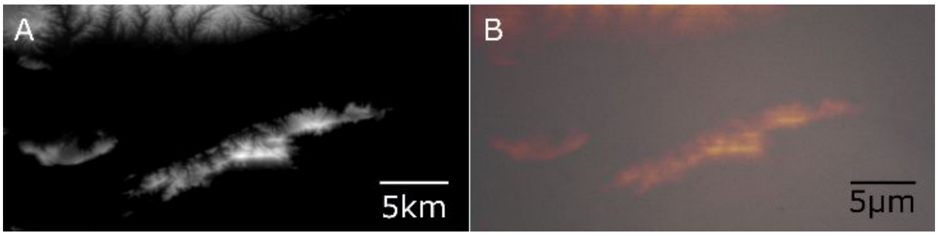

Figure S5: Complex pattern transfer to PDMS according to the procedure in S4. **A)** Original topography map. **B)** Optical micrograph of transferred topography map to PDMS
